# Analysis of Water Coupling in Inelastic Neutron Spectra of Uranyl Fluoride

**DOI:** 10.1038/s41598-019-46675-x

**Published:** 2019-07-19

**Authors:** Andrew Miskowiec, J. L. Niedziela, Marie C. Kirkegaard, Ashley E. Shields

**Affiliations:** 1 Oak Ridge National Laboratory, Oak Ridge, TN 37831 USA; 2 University of Tennessee, Knoxville, TN 37996 USA

**Keywords:** Structure of solids and liquids, Solid-state chemistry, Electronic structure, Chemical physics

## Abstract

Inelastic neutron scattering (INS) is uniquely sensitive to hydrogen due to its comparatively large thermal neutron scattering cross-section (82 b). Consequently, the inclusion of water in real samples presents significant challenges to INS data analysis due directly to the scattering strength of hydrogen. Here, we investigate uranyl fluoride (UO_2_F_2_) with inelastic neutron scattering. UO_2_F_2_ is the hydrolysis product of uranium hexafluoride (UF_6_), and is a hygroscopic, uranyl-ion containing particulate. Raman spectral signatures are commonly used for inferential understanding of the chemical environment for the uranyl ion in UO_2_F_2_, but no direct measurement of the influence of absorbed water molecules on the overall lattice dynamics has been performed until now. To deconvolute the influence of waters on the observed INS spectra, we use density functional theory with full spectral modeling to separate lattice motion from water coupling. In particular, we present a careful and novel analysis of the Q-dependent Debye–Waller factor, allowing us to separate spectral contributions by mass, which reveals preferential water coupling to the uranyl stretching vibrations. Coupled with the detailed partial phonon densities of states calculated via DFT, we infer the probable adsorption locations of interlayer waters. We explain that a common spectral feature in Raman spectra of uranyl fluoride originates from the interaction of water molecules with the uranyl ion based on this analysis. The Debye–Waller analysis is applicable to all INS spectra and could be used to identify light element contributions in other systems.

## Introduction

Fluorinated uranium compounds are ubiquitous in the nuclear fuel cycle but remain a largely understudied component of actinide chemistry. One such example where chemical understanding is still incomplete is uranyl fluoride (UO_2_F_2_), a byproduct of the exposure of uranium hexafluoride (UF_6_) to water. UF_6_ itself is a highly corrosive and reactive gas, and is the primary chemical form used in enrichment operations for nuclear fuel production. When exposed to atmospheric water vapor UF_6_ converts into uranyl fluoride (UO_2_F_2_), releasing hydrogen fluoride (HF). Recently, we showed that the hygroscopic UO_2_F_2_ adsorbs environmental water molecules in a crystal hydrate form^1^. These waters have a significant effect on spectroscopic signatures, in particular the Raman spectra, but accessing this information quantitatively proves difficult for a number of experimental techniques due to various intrinsic limitations based on cross sections or selection rules for scattering.

UO_2_F_2_ is comprised of layers of uranyl ions hexagonally coordinated by fluoride ligands. At ambient conditions anhydrous uranyl fluoride is a hygroscopic powder that can undergo a hydration reaction resulting in uranyl fluoride hydrate^2^. The transition to the hydrated state can be reversed through annealing under vacuum conditions at 115 °C^1^. The exact locations of retained water molecules in the anhydrous structure were heretofore unknown, but the appearance of stacking disorder in x-ray and neutron diffraction patterns offered tantalizing hints of interlayer waters^2,3^.

The uranyl ion is highly Raman active, with a symmetric stretching vibration around 113 meV in the anhydrous form and around 108 meV in the various hydrate structures^4^. Because it the most intense Raman active mode in UO_2_F_2_ and in most uranyl systems, the symmetric stretching vibration of the uranyl ion alone has been used to identify the underlying chemistry^5,6^. In minerals, the frequency and shape of the symmetric stretch mode is sensitive to the uranyl ligand coordination environment, including to the presence of dispersion bonded water molecules^7–11^. Nevertheless, the precise location and the influence of these remnant water molecules on the broader lattice dynamics has not been well understood.

Water dynamics within non-mineral forms of uranium solids remains at the forefront of our efforts to more fully-understand solid-phase uranium chemistry of the nuclear fuel cycle. These efforts are motivated by the need to improve analytical spectroscopic methods for the identification and characterization of non-ideal, and otherwise degraded, fuel cycle solids. The goal of this investigation is to identify the existence and understand the influence of nonremovable water on the lattice dynamics in uranyl fluoride. To achieve this, we combine inelastic neutron scattering (INS) on the *in*-*situ* vacuum-annealed anhydrous structure with density functional theory (DFT) calculations. We expect the total water content to be small in the anhydrous structure such that the observed neutron scattering spectra contain motion of both light and heavy nuclei. Further, unlike in Raman or infrared scattering, there are no selection rules governing scattering of neutrons from the phonons of the UO_2_F_2_ host lattice, allowing collection of a more complete picture of the dynamics. Nevertheless, H atoms coupled to the lattice dynamics complicate analysis, because there is no intrinsic mechanism for separating contributions in INS by mass. To tackle this problem, we offer a novel mechanism to analyze neutron scattering spectra based on application of the equipartition theorem to directly identify motion of adsorbed water in the UO_2_F_2_ structure. We further employ a self-consistent procedure for extracting the phonon density of states from the measured data enabling direct comparison to the phonon density of states from DFT^12–14^. These combined approaches allow us for the first time, to directly ascertain the dynamic affects of the adhered water in the anhydrous uranyl fluoride. The new analysis technique presented here is directly applicable to understanding the water dynamics in a wide variety of materials where residual water is retained in the crystalline structure.

## Materials and Methods

**Figure 1:**
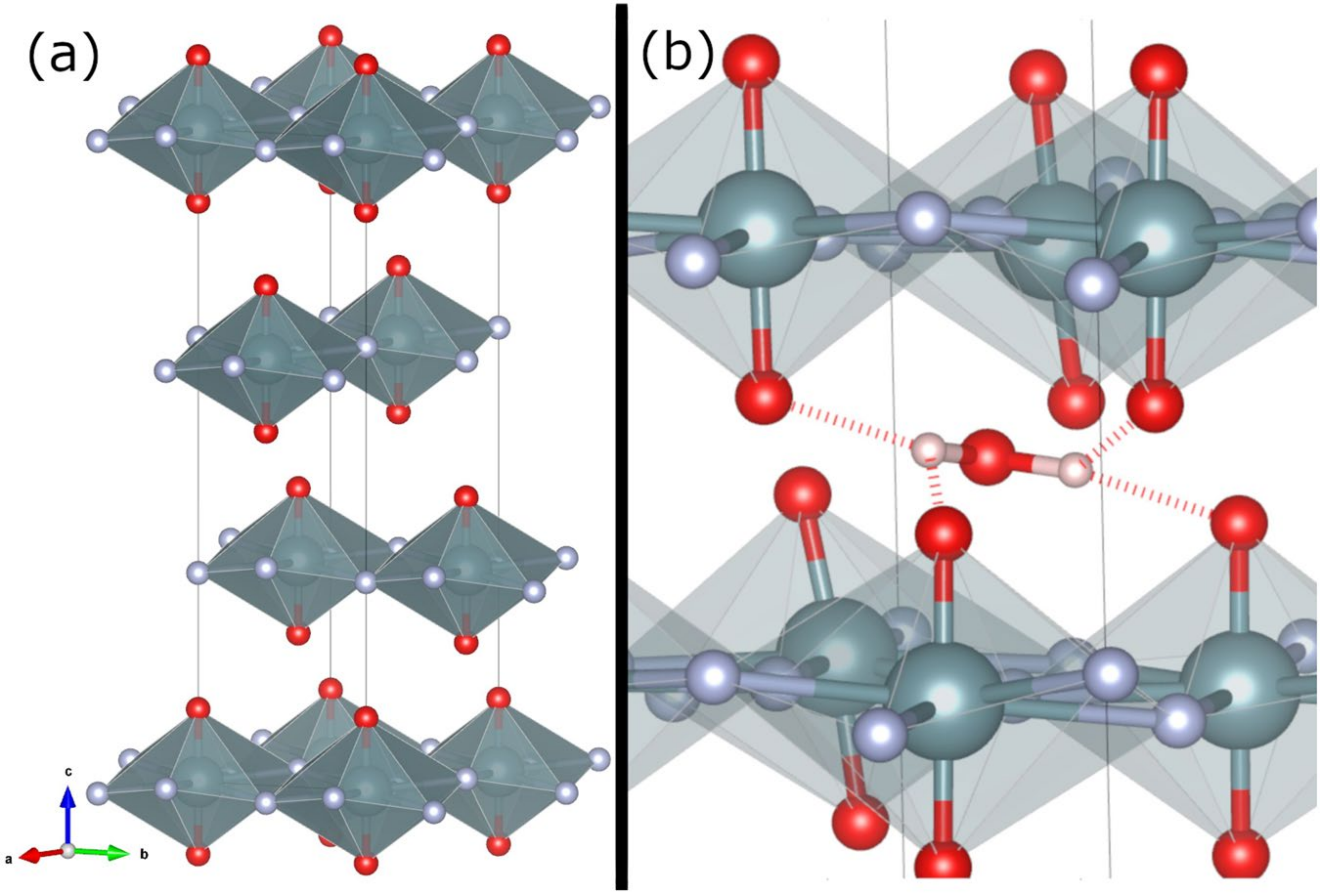
Structure of anhydrous UO_2_F_2_ (U = teal, O = red, F = grey). Panel (a), hexagonal unit cell of UO_2_F_2_ with three-layer repeat structure. Panel (b), relaxed interlayer position of adsorbed water molecule (H = pink), with hydrogen bonds shown. H atoms form strong H-bonds (dashed bonds) with uranyl oxygens (two at 1.81 Å and two at 2.09 Å). Interactions between the water oxygen and neighbor uranyl units result in a O–U–O angle of 165°.

DFT calculations on the anhydrous UO_2_F_2_ structure were previously performed by the authors using the VASP software package^15–19^. Anhydrous UO_2_F_2_ is spacegroup $R\bar{3}m$ (structure shown in Fig. 1); in the hexagonal description, the linear uranyl ion is equatorially coordinated in the hexagonal *a*/*b* plane by six F ligands and stacked in a three-layer repeat motif in the *c* direction. Of particular importance, it was necessary to employ a van der Waals functional to appropriately model the interlayer structure, which is stabilized only by dispersion interactions^20–23^. An on-site Coulomb interaction was also necessary to correctly determine the bond distances and lattice parameters^24,25^. Phonons were calculated using density functional perturbation theory on a 4 × 4 × 4 rhombohedral supercell. A kinetic energy cutoff of 685 eV and a 4 × 4 × 4, ${\rm{\Gamma }}$-centered k-point mesh were used. A Blöchl correction was applied to partial orbital occupations and forces were converged to 0.5 meV/Å^26^. Details regarding these initial calculations can be found in ref.^19^. Here, we are extending those results with calculations of the neutron spectroscopic response. To do this, we used the O’Climax software package^27^. (For details about the production of $S(Q,\omega )$ from calculated phonon eigenvectors, see ref.^27^ and references therein).

Calculations were also performed with a 2 × 2 × 1 hexagonal unit cell containing an adsorbed water molecule. All computational parameters were the same as in the anhydrous structure except that a 2 × 2 × 4, ${\rm{\Gamma }}$-centered k-point mesh was used. Phonon frequencies and eigenvectors were calculated with density functional perturbation theory after geometric relaxation. Figure 1, panel (b), shows the position of a relaxed water molecule, forming H bonds with uranyl oxygens and perturbing the linear O–U–O unit.

INS measurements were collected on the Wide-Angular Range Chopper Spectrometer (ARCS) at SNS^28^. Measurements were collected at 15 K with 20, 80, 175, and 700 meV incident energy neutrons. Neutrons were collected at each incident energy for approximately 4 h for a consistent beam current. Anhydrous uranyl fluoride was prepared by hydrolysis of UF_6_ (UF_6_ + 2H_2_O −> UO_2_F_2_ + 4HF) in a reaction chamber containing approximately 20% relative humidity (23 °C). Uranyl fluoride forms small (<1 *μ*m) particulates from this reaction, which were subsequently collected on a series of silicon plates. The material was baked under a steady flow of 10 mL/s N_2_ at 150 °C for 18 h, a procedure which has been shown to produce anhydrous uranyl fluoride without decomposing the crystal structure^2,29^. The sample was further exposed to vacuum and baked at 150 °C in the beamline for several hours prior to data collection on ARCS to drive off additional surface-adsorbed water. Measurement of the empty, 8 mm diameter Al sample can at the same temperature and incident energies was subtracted from presented data.

## Results and Discussion

**Figure 2:**
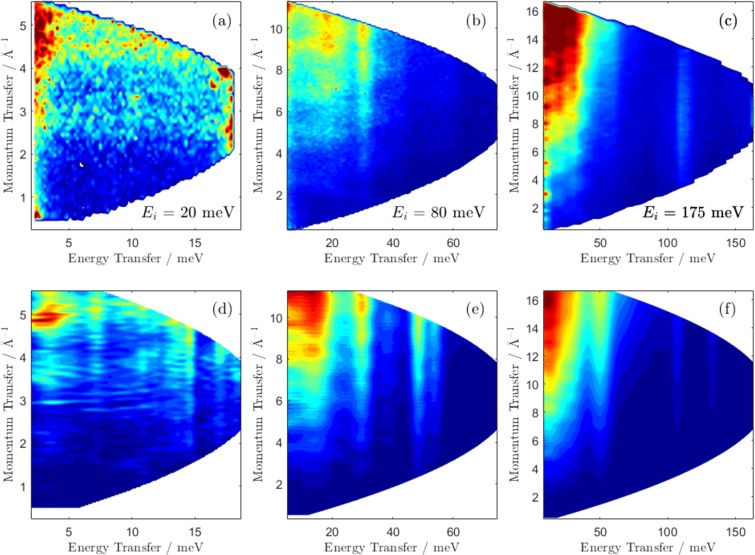
Neutron dynamic structure factor of anhydrous UO_2_F_2_. Top panel: Measured $S(Q,\omega )$ for (**a**) 20, (**b**) 80, and (**c**) 175 meV incident energy neutrons. Bottom panel: Predicted $S(Q,\omega )$ modeled for (**d**) 20, (**e**) 80, and (**f**) 175 meV incident energy neutrons. In the calculation, the estimated energy resolution is modeled according to the known instrument geometry, moderator emission time, and chopper rotation frequencies and a Gaussian broadening is applied. The calculated result is binned in the same binning as the data itself (0.08, 0.1, and 0.2 meV bins), which is narrower than the spectrometer’s energy resolution for the given incident neutron energies (approximately 0.5, 2, and 4 meV at the elastic line for 20, 80, and 175 meV incident energy). Low energy phonon modes are observable below 50 meV, with O–U–O stretching vibrations around 110 meV. The modeled data predicts stronger phonon scattering near 50 meV than is observed, which is related to H coupling as discussed in the text.

In Fig. 2, we plot the background-subtracted neutron dynamic structure factor, $S(Q,\omega )$, as collected on ARCS (top panels) for three incident neutron energies (20, 80, and 175 meV) and a comparison to the same quantity derived from the DFT calculation (bottom panels). The measured phonon spectra contains a low-energy (<50 meV) region containing crystalline phonon modes and an intermediate region near 110 meV with uranyl ion stretching vibrations.

Simulated $S(Q,\omega )$, as produced with the O’Climax code for the same set of incident neutron energies are shown in Fig. 2 panels (d)–(f). O’Climax calculates the instruments’ energy resolution profile given input on the instrument geometry, moderator emission time, and chopper rotation frequencies. It then applies a Gaussian broadening to the points and bins them in 0.08, 0.1, and 0.2 meV bins, which were chosen to match the energy binning in the data sets. The actual energy resolution on ARCS depends on both incident and outgoing neutron energy, but for 20, 80, and 175 meV incident energy neutrons it is approximately 0.5, 2, and 4 meV at the elastic line respectively. Agreement is observed between the calculated and experimental spectra in terms of reproduction of the key peak frequencies. The symmetric and asymmetric uranyl stretching vibrations are observed at 110 and 118 meV. The frequencies of the same vibrations are calculated via DFT to be 110 and 135 meV. The peak frequency at 135 meV disagrees with the data for computational reasons described in Kirkegaard *et al*.^19^. The assignment of these peaks as the uranyl stretching vibrations is unambiguous because the DFT calculation gives the phonon eigenvectors exactly. Observation of coherent phonons is noted with peaks at *Q* = 3.8 and 4.9 Å^−1^ in panel (a) and near 5, 7, and 9 Å^−1^ in panel (b), which is also observed in panels (d) and (e).

**Figure 3:**
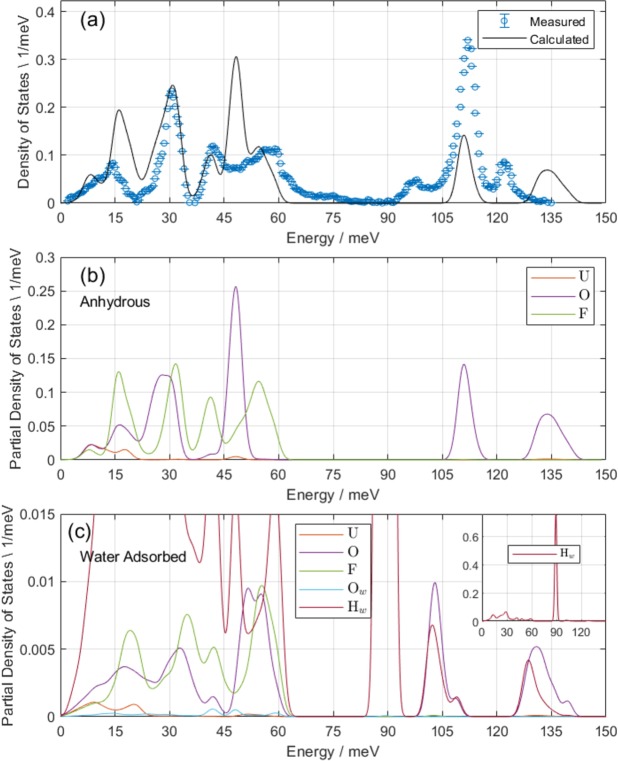
In panel (a), comparison of calculated neutron-weighted (“generalized”) phonon density of states based on phonon eigenvectors and frequencies as calculated via DFT (black line) with measured density of states (blue circles). The calculated values are determined via Equation 1 with a 48 × 48 × 48 k-point mesh sampling the Brillouin zone. The mesh sampling is done via phonopy^31^. Data from measurements of the phonon spectrum with 20, 80, and 175 meV incident energy neutrons are knit together using a self-consistent procedure as implemented in the Mantid data reduction suite and multiphonon package^12–14^. The knitting allows a faithful comparison to the calculated density of states, preserving both the better energy resolution provided by lower energy neutrons and the higher dynamic range of higher energy neutrons. The calculated and measured densities of states agree well with regard to the frequencies of major peaks. The major qualitative differences are a shifting to higher energy and broadening of the 50 meV peak (uranyl bending mode), overestimation of the frequency of the asymmetric uranyl stretching mode (observed at 122 meV), and an underestimation of the intensity of the symmetric uranyl stretching mode. In panel (b), element-decomposed (partial) density of states for U, O, and F atoms as calculated via DFT for the anhydrous structure, shows that a mix of O and F motion dominates below 60 meV, and O motion dominates in the uranyl stretching region. In panel (c), the partial density of states for the water-adsorbed structure shows softening of the 50 meV peak, but due to its large neutron cross-section and small mass, H is the primary contributor to the density of states. The inset in panel (c) shows only H contributions, with a single major peak at 89 meV, corresponding to unhindered librational (*R*_*z*_) motion.

A fundamental quantity derivable from $S(Q,\omega )$ is the generalized phonon density of states (gdos), which can also be determined from the DFT calculation. Because the data is collected at several incident energies, individual data sets must be knit together using a self-consistent procedure to take advantage of both the better energy resolution of lower energy neutrons and the dynamic range of higher energy neutrons. Details of this procedure are outlined in refs^12–14^. In addition to knitting together multiple incident neutron energies, the procedure also corrects for multiple scattering effects, such that the data points in Fig. 3 represent the one-phonon density of states. Generation of this quantity is important because it can be directly compared to the DFT-calculated one-phonon density of states, given by:

1 $${g}_{i,1}(\omega )=\frac{{\sigma }_{i}}{{M}_{i}}\,\sum _{j,Q}\,|{e}_{i}(j,Q){|}^{2}\delta (\omega -\omega (j,Q)),$$

where *σ*_*i*_ is the (total) neutron cross-section of each isotope (with mass *M*_*i*_), ${e}_{i}(j,Q)$ is the phonon eigenvector describing the displacement of each atom along phonon branch *j* at wavevector *Q*, with energy $\omega (j,Q)$^30^. DFT calculations provide both $\omega (j,Q)$ and ${e}_{i}(j,Q)$. Calculation of the full dispersion is done via phonopy with a 48 × 48 × 48 k-point mesh sampling of the Brillouin zone^31^.

Normalization of both curves is done assuming

2 $${\int }_{{E}_{{\rm{\min }}}}^{{E}_{{\rm{\max }}}}\,\sum _{i}\,{g}_{i,1}(\omega )d\omega =3N=15,$$

with *N* representing the number of atoms in the primitive unit cell (5). We select ${E}_{{\rm{\min }}}=0\,{\rm{meV}}$ and ${E}_{{\rm{\max }}}=145\,{\rm{meV}}$, above which there is only molecular scattering. Due to the appearance of additional H atom coupling, this normalization is not correct for the measured data, but nevertheless allows a qualitative comparison to the calculated ${g}_{1}(\omega )$.

We note several key features in Fig. 3, panel (a). First, the position of the main peaks are at 15, 32, 48, 55, 112, and 122 meV. The frequency of the asymmetric uranyl stretching vibration (observed at 122 meV) is overestimated by the calculation, but its assignment is unambiguous. Second, broadening of the experimental gdos is seen near 60 meV, extending up to 80 meV, which is not modeled by the calculation. Third, the modeled data significantly underestimates the gdos in the uranyl stretching region (from 90 to 130 meV). We speculate that this additional scattering intensity in the observed gdos is due to preferential water coupling to the phonon modes involving motion of the uranyl ions, and this is explored in Section 4. Last, the measured gdos is also weaker than the calculated values at the 48 meV peak (*E*_*u*_ uranyl rocking mode, which includes compensatory motion of F ligands) and the 15 meV peak (*A*_1*g*_ describing equatorial F ligand motion along the hexagonal *c* axis).

**Figure 4:**
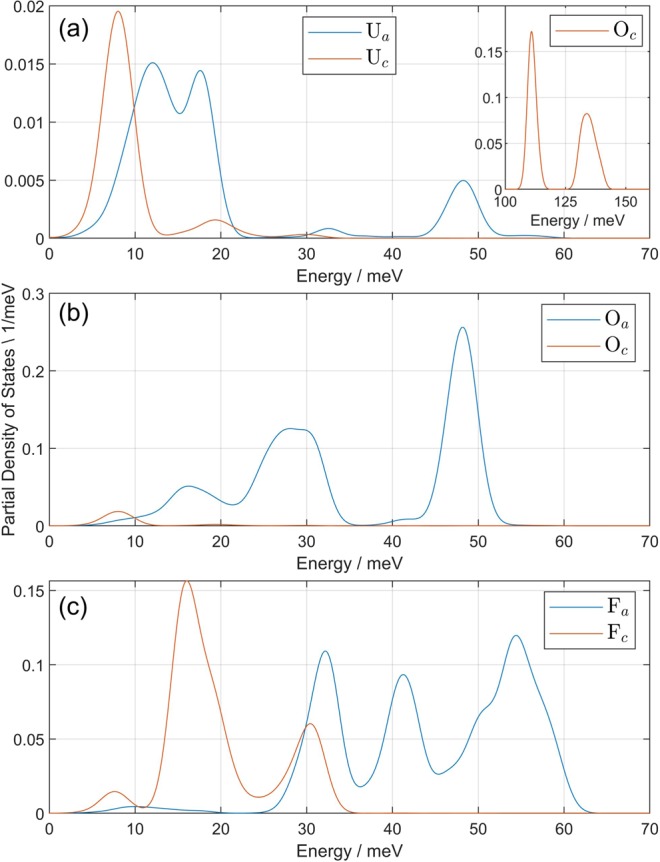
Neutron-weighted density of states projected onto the hexagonal *a*/*b* and *c* axes for U atoms (**a**), O atoms (**b**), and F atoms (**c**). Inset in panel (a) shows density of O atom states projected onto *c* axis for the uranyl stretching region. No fluorine motion is observed in the uranyl stretching region. Uranium motion is primarily restricted to the low energy modes, with exceptions for a bending mode at 48 meV and small contribution to the asymmetric uranyl stretching mode at 133 meV. Due to its large mass, the U atoms contribute little to the full gdos in Fig. 3. Oxygen atoms are distinctly polarized along *a*/*b* below 60 meV and along *c* above 100 meV. Fluorine atoms show the opposite behavior, polarized along *c* at lower energies, but no F motion participates in phonons above 65 meV.

Panel (b) of Fig. 3 shows the partial gdos extracted from DFT calculations. Although the low-energy lattice modes (<70 meV) show a mix of O and F motion, the uranyl stretching region is almost entirely represented by O motion. Motion of U is attenuated by its mass (238 amu) such that its contribution to the measured and calculated gdos is negligible at all energies. Further projection of O and F motion onto hexagonal *a*/*b* and *c* axes in Fig. 4 indicate that the O motions are polarized along *c* at 112 and 122 meV (uranyl stretching region, Fig. 4a inset) and polarized along *a*/*b* below 60 meV (representing primarily uranyl rocking modes). Fluorine motion is predominantly polarized along *a*/*b* for the 42 and 55 meV peaks and along *c* for the 18 meV peak.

Panel (c) of Fig. 3 shows the partial gdos for the structure in Fig. 1(b) with an adsorbed interlayer water molecule. Due to the large neutron cross-section and low mass of H, only one significant peak representing H motion is observed in this region, near 89 meV and is shown in the inset. This phonon mode is a librational mode with H motion polarized along the *a*/*b* axis (*R*_*z*_). We therefore assign the 97.3 meV peak in the data to this mode. Because the observed intensity of the 97 meV mode is low, and it originates from H motion, we further confirm that the quantity of water in this sample is small. Although H is the dominant scatterer in the adsorbed water DFT structure, it is also noteworthy that the O motion near 47 meV is broader and weaker than in the anhydrous case.

**Figure 5:**
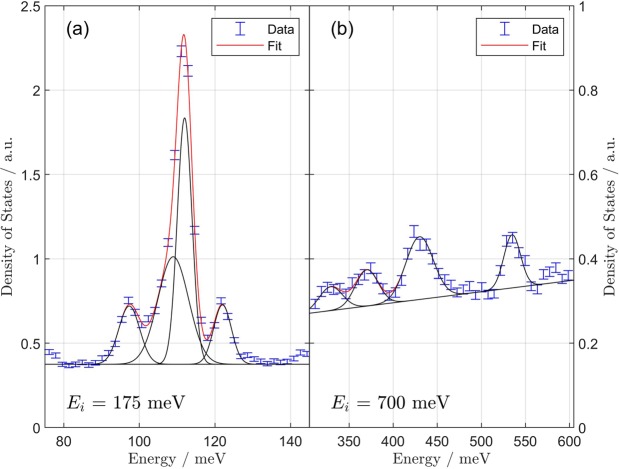
Detailed view of the neutron-weighted (“generalized”) phonon density of states as measured with (**a**) 175 and (**b**) 700 meV incident energy neutrons. The fits in panels (a, b) are a sum of four Gaussian components and a linear background. The fit parameters are provided in Table 1. In panel (a), the symmetric and asymmetric uranyl stretching bands are observed at 112 and 122 meV, respectively. The former also shows a redshift shoulder at 109 meV. The presence of water modes in panel (b) suggests interactions between water and the uranyl ion could be responsible for the redshift shoulder of the symmetric mode. The addition of a coupled water molecule adds 1 u to the effective mass of the O_*yl*_ atom, such that the frequency will be ${\omega }_{{\rm{sym}}+{\rm{H}}}={\omega }_{{\rm{sym}}}\sqrt{\frac{16}{17}}$. For ${\omega }_{{\rm{sym}}}=112.0\,{\rm{meV}}$, ${\omega }_{{\rm{sym}}+{\rm{H}}}=108.6\,{\rm{meV}}$. A similar shift of comparable magnitude should be present for the asymmetric mode, but the intensity of the asymmetric mode is too low to observe this shift. In panel (b), two bands are observed at 370 and 430 meV and can be plausibly assigned to water O–H stretching modes^33^. A weaker band centered at 329 meV is assignable to a combination of H_2_O bending and ${{\rm{UO}}}_{2}^{2+}$ stretching. Likewise, the high frequency band at 535 meV can be attributed to a combination of O–H stretching and ${{\rm{UO}}}_{2}^{2+}$ stretching.

Figure 5 shows a decomposition of the uranyl stretching region and the molecular vibrational region (300–600 meV) into constituent peaks. The uranyl stretching region shown in Fig. 5a decomposes into a set of four Gaussian peaks centered at 97.3, 109.0, 112.0, and 122.0 meV, as enumerated in Table 1. Previous Raman measurements showed that the (Raman-active) symmetric stretching vibration centered at 112 meV manifests a smaller, redshifted shoulder peak^19^. The IR-active asymmetric uranyl stretching peak at 122 meV is in agreement with experiment^32^. A smaller blueshifted shoulder is observed near 129 meV, but because of its weak intensity, we do not assign it *per se*.

**Table 1:** Phonon Density of States Band Assignment and Fitting Parameters. All fits are Gaussian, and widths are FWHM.

Frequency (meV)	Width (meV)	Intensity	Assignment
**Uranyl region**			
97.3 ± 0.2	3.9 ± 0.4	0.35 ± 0.02	*R*_*z*_ H_2_O
109.0 ± 0.6	5.8 ± 0.3	0.64 ± 0.02	${{\rm{UO}}}_{2\,{\rm{sym}}+{\rm{H}}}^{2+}$
111.97 ± 0.04	2.7 ± 0.7	1.46 ± 0.03	${{\rm{UO}}}_{2\,{\rm{sym}}}^{2+}$
122.0 ± 0.2	3.3 ± 0.3	0.35 ± 0.04	${{\rm{UO}}}_{2\,{\rm{asym}}}^{2+}$
**Molecular region**			
212 ± 4	—	—	${\nu }_{2}$ H_2_O
329.2 ± 0.1	18.7 ± 0.4	0.06 ± 0.02	${\nu }_{2}$ ${{\rm{H}}}_{2}{\rm{O}}+{{\rm{UO}}}_{2\,{\rm{sym}}}^{2+}$
370.1 ± 0.1	18.2 ± 0.3	0.09 ± 0.02	O–H stretch
429.9 ± 0.3	22.0 ± 0.7	0.13 ± 0.02	O–H stretch
535.0 ± 0.5	13.4 ± 1.5	0.15 ± 0.03	O–H stretch + ${{\rm{UO}}}_{2\,{\rm{sym}}}^{2+}$

In the molecular region (Fig. 5, panel [b]), peaks can be assigned to O–H stretching modes at 370 and 430 meV as listed in Table 1^33^. The apparent increased background is due to the presence of a hydrogen recoil peak, which adds an incoherent background at higher incident neutron energies but no vibrational information. The existence of higher energy molecular vibrations and a hydrogen recoil peak confirm the presence of water in the experimental sample. As surface-adsorbed water was removed prior to experiment, the remaining water is further evidence that the layered UO_2_F_2_ crystal supports the localization of water from the environment, either during formation or immediately afterwards^1,3^. In Fig. 1(b), H atoms form H bonds with uranyl oxygens at two distinct distances, O–H–O = 2.639 and 2.820 Å. As tabulated by Libowitzky, these distances would correspond to O–H stretching frequencies of about 366 and 425 meV^34^. Thus, both the 370 and 430 meV could correspond to O–H stretches, related to interactions with different uranyl ions. The appearance of multiple O–H stretching frequencies originating from a single water molecule has previously been observed in mineral hydrates such as natrolite and scolecite^35^.

A vibrational peak is observed at 534 meV, which is too high to be assignable to fundamental water vibrations. This peak is likely a combination band (O–H plus the uranyl vibrations at 112 meV). A water bending mode near 220 meV was observed but difficult to assign directly due to relatively weak signal to background. However, a similar O–H bend plus uranyl vibration is observed in Fig. 5 at 329 meV, which allows us to indirectly assign ${\nu }_{2}-{{\rm{H}}}_{2}{\rm{O}}$ to 212 meV.

Finally, we explored two alternative models with hydrogenated species. One, we considered uranyl fluoride with an interlayer OH^−^ ion, and two, a model in which a F ligand were replaced by an OH^−^ ion. Both models failed to reproduce key features of the data; in particular, the OH^−^ ligand model resulted in a complete layer collapse and significant red-shifting of the uranyl stretching frequencies due to formation of interlayer O–H–F hydrogen bonds, whereas the free OH^−^ ion model suggested the appearance of several strong peaks around 65 meV that are not observed.

## Water Coupling

The observed water O–H stretching frequencies are both lower than for liquid water and fall within the range of “strong” hydrogen bonding^34^. In solids, the ${\nu }_{2}$ frequency is shifted to higher energy due to H bonds, which is also observed here despite the larger uncertainty^36^. Observed water fundamental frequencies suggest strong H bonding.

In Fig. 3 the major disagreement between theory and experiment is the mismatch in intensity between the higher energy uranyl stretching region (90–130 meV) compared to the generally good agreement below 90 meV (both frequencies and intensities). The high energy molecular region (Fig. 5 panel [b]) indicates the existence of O–H stretching frequencies, as well as the presence of a hydrogen recoil peak. Thus, despite having been vacuum annealed, anhydrous UO_2_F_2_ contains residual water. Our previous neutron diffraction and quasielastic neutron spectroscopy measurements also indicated the existence of a strongly bound set of water molecules that could not be removed without substantial (>250 °C) annealing that can result in oxidation and damage^1,3^. Those water molecules are an intrinsic part of the structure.

To understand the interaction of water molecules with the lattice modes, we examine the *Q*-dependence of the observed data. The *Q*-dependence of the intensity of phonon bands measured in INS experiments can be described by

3 $$\begin{array}{rcl}S(Q,\omega ) & = & \frac{1}{2N\langle {b}^{2}\rangle }\,\sum _{i,i^{\prime} }\,{b}_{i}{b}_{i^{\prime} }\,\exp (\,-\,{W}_{i}(Q)-{W}_{i^{\prime} }(Q))\\  &  & \times \,\sum _{j}\,\frac{(Q\cdot {e}_{i}^{j})(Q\cdot {e}_{i^{\prime} }^{j})}{\sqrt{{M}_{i}{M}_{i^{\prime} }}{\omega }_{j}}\\  &  & \times \,({n}_{j}+1)\,\exp (iQ\cdot ({R}_{i^{\prime} }-{R}_{i}))\delta (\omega -{\omega }_{j}),\end{array}$$

where *b*_*i*_ is the scattering length for isotope *i* and *n*_*j*_ is the thermal population factor of mode *j*, with frequency ${\omega }_{j}$, ${e}_{i}^{j}$ is the eigenvector describing the displacement of atom *i* for mode *j*, *M*_*i*_ are the masses of species atom *i*, *R*_*i*_ is the position of atom *i*, and *N* is the number of atoms^37^. The ${W}_{i}(Q)$ are the Debye–Waller factors, which are

4 $$W(Q)=\langle {u}^{2}\rangle {Q}^{2}.$$

**Figure 6:**
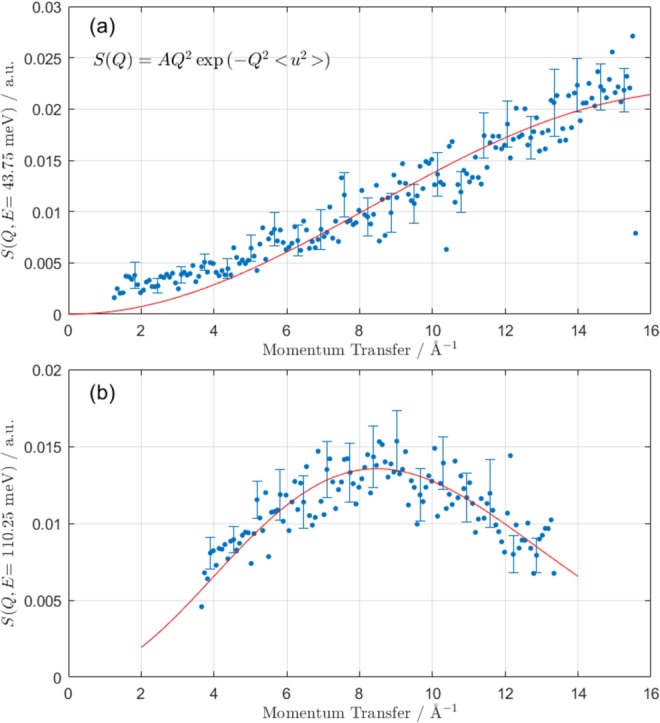
Example *S*(*Q*) for two particular energy cuts (panel (a), 43.00–44.50 meV and panel (b), 109.50–111.00 mev), with fit to Equation 5. Qualitatively, *S*(*Q*) in panel (b) is attenuated more strongly in *Q* due to the larger Debye-Waller factor of the atoms participating in motion at that frequency.

The mean-square displacement of atoms from their equilibrium positions is $\langle {u}^{2}\rangle$. In a powder average, incoherent approximation, Equation 3 can be simplified:

5 $$S(Q,\omega )=A(\omega ){Q}^{2}\,\exp (\,-\,\langle {u}^{2}\rangle {Q}^{2}),$$

where $A(\omega )$ contains all information related to the intensity as a function of frequency (the scattering lengths, polarization average, thermal factor, and frequency denominator). The $S(Q,\omega )$ experimental data are binned in 1.5 meV bins and fit to Equation 5 with two free parameters (*A* and *u*^2^). The critical observation in this system is the clear difference in *Q*-dependence of different peaks as shown in Fig. 2. The mode at 112 meV (uranyl stretching vibration) is peaked near 9.8 Å^−1^, whereas the 32 meV mode is centered at 16.2 Å^−1^. Two examples of this *Q*-dependence, as well as their fit to Equation 5, are shown in Fig. 6, which clearly shows a sharper attenuation in *Q* for the higher energy uranyl stretching mode region. For a given $\omega$, only certain *Q* values are accessible based on instrument geometry and neutron kinematics, but data from all accessible *Q* values for each energy bin is included in each fit. The mean-square displacement, $\langle {u}^{2}\rangle$, is inversely proportional to mass as a requirement of the equipartition theorem^38^. As a consequence, modes involving significant contributions from H atoms will demonstrate stronger *Q* attenuation.

**Figure 7:**
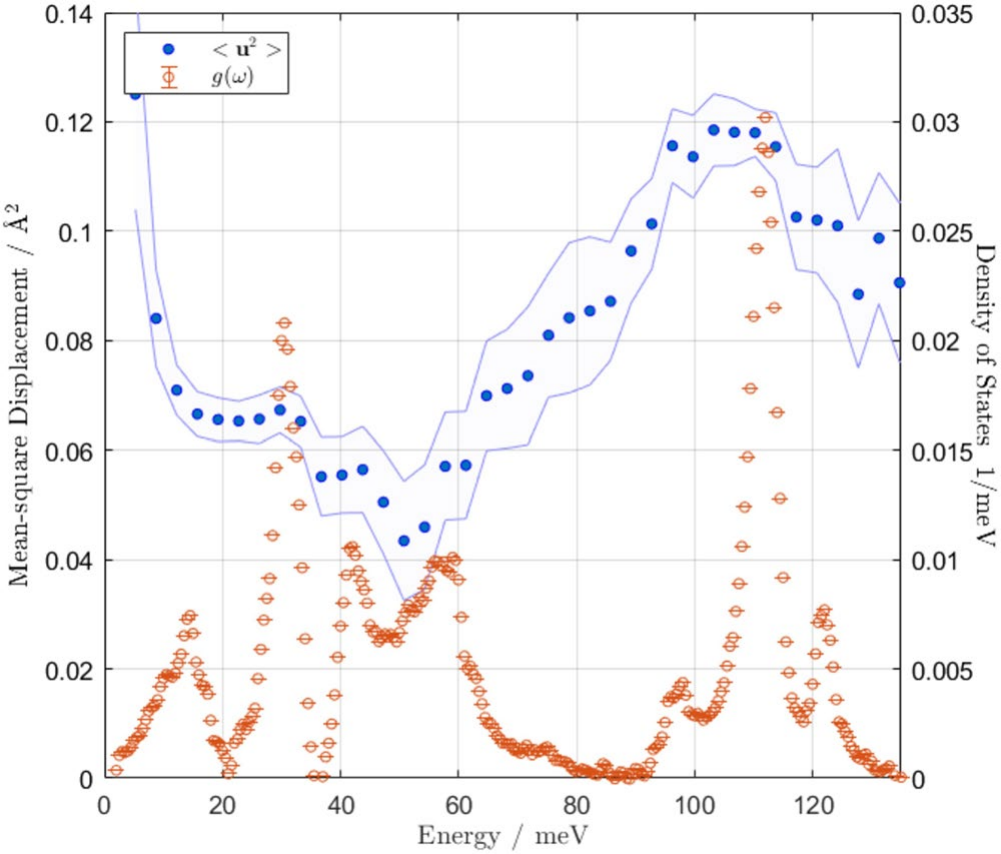
Calculated mean-square displacement (Debye–Waller factor) as a function of energy for 175 meV incident energy neutrons. The blue points (left axis) are calculated by fitting binned regions of $S(Q,\omega )$ (Fig. 2) to Equation 5. The error channel represents a 95% confidence interval. The tan points (right axis) are the reproduced experimental gdos (Fig. 3) for comparison. The mean-square displacement parameter is responsible for attenuation of scattered intensity as a function of *Q*. Since hydrogen is much lighter than the other sample constituent elements, the same kinetic energy is associated with a larger mean displacement from equilibrium. By identifying regions of larger mean-square displacement in the phonon spectrum, we identify regions of stronger H-coupling to the lattice. In particular, the uranyl stretching region (90–130 meV) shows the strongest *Q* attenuation in $S(Q,\omega )$ and the strongest mismatch between the calculated and measured gdos (Fig. 3), both indicative of significant H-coupling.

In Fig. 7 we fit $S(Q,\omega )$ from Fig. 2 to Equation 5, by binning $S(Q,\omega )$ into 1.5 meV slices and extracting $\langle {u}^{2}\rangle$ for each slice. For reference, we include the previously reported neutron-weighted density of states from Fig. 3. The strongest *Q* attenuation (i.e., highest Debye–Waller factor) is observed in the uranyl stretching region (90–130 meV), indicating that the observed data represent constituents with larger mean-square displacements (H). Cross-referencing this region with Fig. 4, panel (b), we note that the mode decomposition for the uranyl stretching modes is nearly entirely the motion of the O atoms along the crystallographic *c* axis.

Water couples most strongly to the uranyl vibrations in UO_2_F_2_ based on the following pieces of evidence: One, the calculated gdos (which includes only U, O, and F motions) is much lower than the observed gdos in the uranyl stretching region, indicating a strong scatterer at the same frequency (H) is present; two, significant *Q* attenuation is noted as per Fig. 7 selectively for the 90–130 meV uranyl stretching region; three, the computed partial phonon gdos in Fig. 3, panel (b) reveals that the uranyl oxygens dominate atomic motion at those frequencies, disambiguating the assignment of those modes to uranyl stretches and four, there is a significant density of vibrational states of O–H stretching plus O–U–O stretching combination bands in the observed gdos.

Because of the layered nature of the anhydrous UO_2_F_2_ structure, it is logical that the adsorbed water molecules will form H bonds with the uranyl oxygens. The above evidence indicates that this intuition is correct. A possible position of adsorbed water molecules is shown in Fig. 1, panel (b), as relaxed with DFT. This interlayer water position accommodates between two and four H-bonds with neighboring uranyl ions and fills the space between interlayer equatorial F ligands (separated by 5.6 Å). With respect to the attenuation of the measured gdos compared to the calculated gdos at 48 and 15 meV (Fig. 3(a)), recall the resolution of the partial density of states into components of motion (Fig. 4) indicate that the motion at 15 meV is entirely F atoms moving along the hexagonal *c* axis, and the motion at 48 meV is primarily motion of O atoms moving along the *a*/*b* axes. We observe that both of these atomic motions collide with the location of adsorbed water molecules, attenuating the gdos by a mechanism of steric hindrance^39–41^. In particular, the effective oscillator mass is increased by the coupling of O/F atoms to the H motion, reducing the expected scattering power. Previous INS measurements also identified signatures of this effect in the mean-square displacement^42,43^. Whereas, the atomic motions of the uranyl oxygens in the uranyl stretching region (strong H coupling) are polarized along *c* and not in collision with adsorbed waters. In short, a vector drawn between uranyl oxygens or ligand F atoms to the proposed water position in Fig. 1 panel (b) is collinear with the phonon polarization at the same frequencies where we observe additional attenuation in the gdos. The effect of the additional water molecule on the gdos clearly corroborates this interpretation in Fig. 3, panel (c), because the 15 and 48 meV modes are preferentially attenuated compared to the same modes in the anhydrous structure, and significant water H motion is present at those frequencies.

According to the inset in Fig. 3, panel (c), the primary observable in the gdos for adsorbed water molecules is the appearance of the *R*_*z*_ librational peak. The observation of this mode in the data is strong evidence for the presence of interlayer water, especially because the anhydrous UO_2_F_2_ structure has no vibrational modes to explain this mode. The model structure also *directly* shows coupling between adsorbed water molecules and the uranyl vibrations as evidenced by the overlap of gdos between uranyl O and water H atoms.

In Table 1 we identify two peaks at 109 and 112 meV associated with the symmetric stretching mode of the uranyl ion. Raman spectra of the same vibrational mode consistently show a similar two peak decomposition, always with a redshift shoulder^1,4,44^. For the symmetric mode, the vibrational frequency is proportional to the square root of the oscillator mass (16 amu for O). The existence of transient O_*yl*_–H bonds increases the effective mass of the oscillator by 1 amu. Considering most uranyl ions are not near water molecules (as required by previous thermal gravimetric measurements), the fundamental oscillation frequency at 112 meV represents unperturbed uranyl ions^2^. Adding a nearby H-bonded water molecule shifts the frequency by

6 $$\begin{array}{rcl}{\omega }_{{\rm{sym}}+{\rm{H}}} & = & {\omega }_{{\rm{sym}}}\sqrt{\frac{{m}_{O}}{{m}_{O}+{m}_{H}}}\\  & = & 111.98\sqrt{\frac{16}{17}}{\rm{meV}}=108.64\,{\rm{meV}}\end{array}$$

compared to the measured value of 109.0 meV. This phenomenon has been observed in a variety of other water-containing uranyl species, such as uranyl hydroxide, ammonium diuranate, ammonium uranyl carbonate, sodium diuranate, and uranyl acetate^45–47^. This mechanism of H-bonding damping may be responsible for the line shape of uranyl stretching vibrations. Redshift in the uranyl stretching vibrations in Fig. 3, panel (c), due to the presence of water corroborates this hypothesis. More investigation, such as careful isotopic substitution, could further improve the interpretive value of this commonly used vibrational signature. Previously, we attempted to equilibrate samples of hydrated UO_2_F_2_ with deuterated saturated salts, but INS measurements indicated only a fraction of water molecules were exchangeable^1^. Entrainment of D_2_O into UO_2_F_2_ may thus require formation in a D_2_O environment.

## Conclusions

Using a combination of INS and DFT modeling, the vibrational spectra of anhydrous UO_2_F_2_ was explored. An enhancement of scattering intensity in the 90–130 meV uranyl stretching region compared to the modeled intensity suggested the presence of a strong scatterer, which was confirmed by the appearance of O–H stretching modes in the molecular vibrational region (>350 meV) and the appearance of a water librational mode at 97 meV. Observation of H atoms in anhydrous UO_2_F_2_ corroborated previous investigations that postulated the existence of strongly bound water^1^.

Detailed analysis of the *Q*-dependence of the intensity of $S(Q,\omega )$ indicates preferential attenuation of intensity in the uranyl stretching region. Correlation of this attenuation with oxygen motion (indicated by the partial phonon gdos), as well as the observed scattering intensity enhancement in that region, indicates a strong and preferential coupling between adsorbed, nonremovable water molecules and the uranyl stretching vibrations (particularly the symmetric stretching mode). Steric hindrance between adsorbed water molecules and the backbone structure, as indicated by the attenuation of certain vibrational modes whose atomic motions collide with the locations of those adsorbed waters, independently substantiate this argument.

## Data Availability

Data sets and DFT input files can be made available upon reasonable request to the corresponding author.
